# Does self reflection and insight correlate with academic performance in medical students?

**DOI:** 10.1186/1472-6920-13-113

**Published:** 2013-08-23

**Authors:** Sandra E Carr, Paula H Johnson

**Affiliations:** 1 Education Centre, Faculty of Medicine, Dentistry and Health Science, University of Western Australia, M515, 35 Stirling Highway, Nedlands, WA 6009, Australia; 2 School of Medicine and Pharmacology Fremantle Hospital Unit, Faculty of Medicine Dentistry and Health Science, University of Western Australia, PO Box 480, Fremantle WA 6959, Australia

**Keywords:** Self reflection, Insight, Medical students

## Abstract

**Background:**

Medical students in academic difficulty are often described as lacking insight. The Self Reflection and Insight Scale (SRIS) is a tool for measuring insight which has been validated in medical students. We investigated whether self reflection and insight scores correlate with academic performance in Year 4 medical students from a six year undergraduate medical degree, and whether self reflection and insight changes after one year of clinical training.

**Methods:**

Self reflection and insight scores were measured in 162 students at the start of Year 4 at the University of Western Australia. Performance in end of year written and clinical exams was monitored and correlated with SRIS. Seventy of the students were surveyed again at the start of Year 5 to see if scores changed or were stable after one year of full time clinical training.

**Results:**

We found no correlation between self reflection or insight and academic performance in written and clinical exams. There was a significant increase in recognition of the need for self reflection in Year 5 compared with Year 4.

**Conclusions:**

While no correlation was found between this measure of self reflection and insight with academic performance, there was an increase in students’ recognition of the need for reflection after one year of clinical studies. This study is a valuable first step towards a potentially exciting research domain and warrants further longitudinal evaluation with larger cohorts of students using additional measures of achievement.

## Background

Central to the practice of medicine are the concepts of self regulation and a commitment to life long learning. Medical students are now expected to undertake much of their learning in the form of self-directed study rather than being the passive recipients of didactic teaching. In order for self directed learning and self regulation to be effective the student (or doctor) needs to have a degree of awareness of their own knowledge and performance, often referred to as *Insight*[1]. Self-regulation requires a professional to have the capacity for change when presented with evidence of suboptimal performance and insight is necessary for this process [1]. There is evidence to show that poorly performing medical students lack insight into the reasons for their suboptimal performance [2,3] and that some forms of poor performance may not be amenable to remediation [3,4].

A lack of insight is of particular concern in medical students and is often cited anecdotally by their supervisors as the reason for failure to improve after remediation [3]. Medical students now train in a world in which the professional performance of doctors is coming under increasing scrutiny by medical boards, the media and patients [5]. This has led to a major shift in how medical schools teach and monitor standards of professional behaviour in their students. Medical schools across the world have introduced formal methods of monitoring and remediating students who display behaviour considered to constitute poor professional performance [6]– [8]. Some medical boards have published guidelines specifically for students detailing the professional behaviours expected of them [9].

Insight has until recently been a quality which, although understood well by teachers and clinicians (particularly when lacking) has been difficult to measure objectively. While there is limited literature surrounding this area in medical education a scale for measurement of insight has now been published and validated in medical students [10]. This has provided an opportunity to measure insight in a cohort of students.

The aims of this project were as follows: to compare self reflection and insight of clinical medical students in our institution with those reported by the authors of the SRIS [10]; to explore prospectively whether self reflection and insight correlated with academic performance in end of year written and clinical exams and to explore whether SRIS scores changed during the first year of clinical training in our students.

## Methods

The Self Reflection and Insight Scale (SRIS) [10] is a 5 point Likert scale type questionnaire asking subjects the extent to which they agree or disagree with 20 statements. The responses to each question are scored on a scale of one to five with one equating to “strongly disagree” and five to “strongly agree”. The statements relate to three domains of insight: recognition of the need for reflection, the process of engaging in reflection and the presence of insight. Roberts and Stark validated the SRIS with medical students using a factor analysis [10] that showed all items loading significantly onto the three expected factors with a good fit to the data. The identified subscales demonstrated good internal reliability (> 0.8) [10]. The same three subscales have been used in this study to facilitate comparison with previously published research. The SRIS is provided as Additional file 1 (PDF format).

A total score for each component of the questionnaire is calculated as follows:

a) Engaging in self-reflection (items 7,12,18,2,15,5) maximum possible score 30.

b) Need for self-reflection (items 8,16,1,19,10,13) maximum possible score 30.

c) Insight (items 17,14,11,4,9,20,6,3) maximum possible score 40.

### Data collection

In 2009, all 200 Year 4 medical students of a six year undergraduate course at the University of Western Australia were asked to complete the SRIS while in a large group setting on the first day of the academic year. Ethics approval for the study was obtained from the Human Research and Ethics Committee of the University of Western Australia.

The students gave consent for their insight and self reflection scores to be correlated with two measures of academic performance:

1. Mark in end of Year 4 integrated written examination assessing knowledge for the discipline areas of surgery, medicine and psychiatry.

2. Mark in end of Year 4 Objective Structured Clinical Examination (OSCE). This assesses the knowledge and skills required for generic patient assessment and management. The participating disciplines are surgery, medicine, psychiatry and infectious diseases.

In addition the SRIS was re-administered to the same cohort 12 months later at the commencement of Year 5 to explore changes in the cohort insight and self-reflection scores over time.

### Data analysis

Questionnaires were excluded if respondents left items unanswered so that component scores could not be calculated. All coded data for the respondents agreeing to participate in the study was entered into SPSS 17.0®. Descriptive statistics were used to report the participants’ scores for each item, in each of the three branches relating to three components of:and for overall reflection scores (engaging plus need for reflection combined). A one way analysis of variance was performed to examine correlations between the self reflection and insight scores with the performance measures. Differences in participants’ scores from Year 4 to Year 5 were analysed through paired sample correlations for the three components and the overall reflection scores. A Bonferroni post hoc correction was applied to control for the problem of increased false positive results observed when multiple comparisons are being made. This correction resulted in a p value of 0.01 being applied as the required level for significance.

a) Engaging in self-reflection,

b) Need for self-reflection and

c) Insight

## Results

One hundred and sixty two of the 200 (81%) students completed the survey at the beginning of Year 4. Of these 92 (57%) were female and 70 (43%) were male which was representative of the gender distribution of the whole cohort. The mean age was 25 years. Of the 162 respondents at the beginning of Year 4, 70 (43%) also completed the SRIS survey at the beginning of Year 5 with 40 (57%) respondents being female and 30 (43%) male. The range and distribution of scores in the subgroup of 70 students in Year 4 compared with the total respondent population of 162 Year 4 students did not differ. This indicates that the scores in this subgroup were representative of the Year 4 medical students.

Table 1 shows the scores for each component of the SRIS for students at the start of Year 4 and Year 5. There were no significant differences in scores for any component between male and female respondents at the Year 4 or Year 5 level. Subsequent analyses did not look for differences between gender.

**Table 1:** Descriptive statistics for the components of the self reflection and insight scale (SRIS) for 162 Year 4 students and the 70 students who repeated the SRIS in year 5

**Component (total possible score)**	Year 4 N*	**Mean score (SD)**	Year 5 N*	**Mean score (SD)**
Engage in reflection (/30)	162	17.0 (2.2)	68	16.5 (2.0)
Need for reflection (/30)	162	20.2 (3.9)	70	23.5 (3.5)
Self insight (/40)	161	19.4 (2.9)	69	18.9 (3.1)
Overall critical self-reflection (/60)	162	37.2 (4.8)	68	40.1 (4.5)

*Questionnaires were excluded if students left items uncompleted and a total component score could not be calculated.

Table 2 shows the correlations between the SRIS component scores and measures of academic performance at the end of Year 4. No significant correlations were found when SRIS component scores were compared with student performance in the Year 4 written exam and the OSCE results.

**Table 2:** Correlations of the self reflection and insight scale (SRIS) component scores with performance measures in year 4

**Performance measure**		**Written exam**	**OSCE**	**Engage in reflection**	**Need for reflection**	**Self insight**
**Written Exam**	Pearson Correlation	1	.28^**^	.09	-.09	-.12
	Sig. (2-tailed)		.00	.30	.26	.14
	N	162	181	155	155	155
**OSCE**	Pearson Correlation	.28^**^	1	.13	.08	-.07
	Sig. (2-tailed)	.00		.14	.32	.40
	N	162	185	145	145	144

**Correlation is significant at the level of 0.01.

Table 3 shows the comparison of the SRIS scores between Year 4 and Year 5 just for the 70 students who completed the SRIS twice.

**Table 3:** Paired sample mean scores for self reflection and insight scale (SRIS) component scores from Year 4 to Year 5

**Component (total possible score)**	**Year 4**	**Year 5**	**95% CI of the difference in the paired sample mean scores (Yr 4- Yr 5)**		**Significance**
	**(n = 70)**	**(n = 70)**			
	**Mean (SD)**	**Mean (SD)**	**Lower**	**Upper**	
Engage in reflection (/30)	17.5 (2.2)	16.5 (2.0)	.24	1.70	.010**
Need for reflection (/30)	20.6 (2.7)	23.5 (3.5)	−3.77	−1.95	.000**
Self insight (/40)	19.5 (2.9)	18.9 (3.1)	-.31	1.42	.201
Overall critical self-reflection (/60)	38.2 (4.1)	40.1 (4.5)	−3.23	-.57	.006**

**p < 0.01.

## Discussion

Our study is the first to report sequential scores for self reflection and insight in the same group of students from the beginning of one academic year to the next.

The original study validating the SRIS found scores to be slightly higher in males which we did not replicate. There are two possible reasons for this difference, which was small, in the report by Roberts et al. [10]. Either our sample size was not big enough to detect a difference (as we had 162 in our study and Roberts et al. had a sample size of 462) or the reported difference in the original study was spurious.

Secondly our mean SRIS scores were lower than those reported by Roberts et al. [10]. There are several possible explanations for this. Differences in course structure could have accounted for the difference. Our course is a traditional Australian six year course with little clinical contact in years 1 to 3 and full time clinical training starting in Year 4. Both undergraduate and postgraduate entry students come together in Year 4 on our course to start clinical training. Roberts et al. [10] studied students in the UK on a new five year undergraduate course and it is not clear from their description whether their students have substantial clinical contact from Year 1 of their course. Different selection methods for the courses may also provide an explanation. It is possible that different interview techniques could select candidates with attributes which score higher or lower on the SRIS. Roberts et al. [10] found that insight increased with age in their cohort but this does not explain the differences between their results and ours as the mean age of our students (at 25 years) was higher than the cohort studied by Roberts et al. (mean age 22 years) [10].

Thirdly our finding of an increase in Year 5 compared with Year 4 in measured need for self reflection but a reduction in engagement in reflection is intriguing. Our measured increase in overall critical self reflection could be accounted for by the increase in scores for the component need for self reflection. Our measured increase in need for self reflection from Year 4 to Year 5 could be explained by themes running in Year 4 of the course: our students have a Personal and Professional Development unit running throughout the clinical years of the course. Tutorials on reflective practice are run in Year 4 and this may have had an influence on SRIS scores leading to a rise at the start of Year 5. It is also possible that an increase in this score is the “natural result” of exposure to the first full year of clinical practice on our course. The reason for the reduction in engagement in reflection is difficult to explain and the educational significance of this finding is unclear but concerning if it is not artifact. This finding warrants further investigation through qualitative methods such as focus groups and interviews.

Lastly the lack of correlation of SRIS scores with the academic outcomes of examination results raises a number of points for discussion. The Year 4 OSCE is designed to test generic clinical competence and not insight. Similarly the SRIS is not a test of clinical competence. Our postulation that *SRIS = true insight = competence* may be too simplistic or frankly incorrect. Although some studies [2,3] and plenty of anecdotal experience suggest that lack of insight goes hand in hand with poor practice, it may not follow that good insight leads to clinical competence. It is also possible that the SRIS may not be sophisticated enough to measure “insight” in the sense that is understood and described by clinicians dealing with poorly performing students and junior colleagues.

There are very few studies comparing measures of self-reflection with academic performance in medical students or related disciplines. Lew and Schmidt [11] studied self-reflection in 690 young (average age 17 years) first year applied science students in Singapore, finding a weak correlation between quality of reflection journal writing and academic performance. Stephens et al. [12] looked at multiple measures of academic performance in 994 medical students in one institution in the USA. All students took a first year course on the “Human context of health care” incorporating a graded reflective writing essay, and a correlation was observed at the end of the course between the grades for this essay and overall grade point average for the whole degree. Burnett et al. [13] assessed reflective accounts written by final year medical students in the context of the introduction of a hand hygiene unit. They found high inter-rater agreement for the validated assessment instrument used, but did not follow the cohort into clinical practice to assess any correlation with hand hygiene behaviour after graduation. Comparing studies is problematic owing to the different tools used to assess self-reflection and academic performance in each institution. In addition our study attempted to measure insight and self reflection which may be subtly more complex than measuring the ability to reflect, thereby making meaningful comparison with studies measuring reflective ability difficult.

## Conclusions

This study is ongoing with a plan to follow this cohort of students through to the commencement of their final year of the medical course to explore the further development of self reflection and insight skills. This study is a valuable first step towards a potentially exciting research domain and warrants further longitudinal evaluation with larger cohorts of students using additional measures of achievement.

## Abbreviations

OSCE: Objective structured clinical examination; SRIS: Self reflection and insight scale.

## Competing interests

The authors declare that they have no competing interests.

## Authors’ contributions

SC contributed to the design of the study, contributed to data collection, performed the statistical analysis and edited the manuscript. PJ contributed to the design of the study, contributed to data collection and drafted the manuscript. All authors read and approved the final manuscript.

## Pre-publication history

The pre-publication history for this paper can be accessed here:

http://www.biomedcentral.com/1472-6920/13/113/prepub

## Supplementary Material

Additional file 1 The Self reflection and insight scale (SRIS). Click here for file
