# Supplementary material for: Does self reflection and insight correlate with academic performance in medical students?
Source: BMC Med Educ. 2013 Aug 23;13:113. doi: 10.1186/1472-6920-13-113 (PMC3765283; doi:10.1186/1472-6920-13-113)
Supplement: Additional file 1 — The Self reflection and insight scale (SRIS). [file 1472-6920-13-113-S1.pdf]

Appendix 1: The Self reflection and insight scale (SRIS)

**Self reflection and insight questionnaire: subjects respond on a likert scale as follows: "Strongly agree, agree, neutral, disagree, strongly disagree"**	
1	I don't often think about my thoughts [1]
2	I am not really interested in analyzing my behaviour [2]
3	I am usually aware of my thoughts [3]
4	I am often confused about the way that I really feel about things [3]
5	It is important for me to evaluate the things that I do [2]
6	I usually have a very clear idea about why I have behaved in a certain way [3]
7	I am very interested in examining what I think about [2]
8	I rarely spend time in self reflection [1]
9	I'm often aware that I am having a feeling, but I often don't quite know what it is [3]
10	I frequently examine my feelings [1]
11	My behaviour often puzzles me [3]
12	It is important to me to try to understand what my feelings mean [2]
13	I don't really think about why I behave in the way that I do [1]
14	Thinking about my thoughts make me more confused [3]
15	I have a definite need to understand the way my mind works [2]
16	I frequently take time to reflect on my thoughts [1]
17	Often I find it difficult to make sense of the way I feel about things [3]
18	It is important to me to be able to understand how my thoughts arise [2]
19	I often think about the way I feel about things [1]
20	I usually know why I feel the way I do [3]

Items 1,2,4,8,11,13,14 and 17 are reversed.

1. Component a) Engaging in self-reflection

2. Component b) Need for self-reflection

3. Component c) Insight
